# An Assessment of the Nutritional Value of the Preschool Food Rations for Children from the Wroclaw District, Poland—The Case of a Big City

**DOI:** 10.3390/nu14030460

**Published:** 2022-01-20

**Authors:** Agnieszka Orkusz

**Affiliations:** Department of Biotechnology and Food Analysis, Wroclaw University of Economics and Business, 53-345 Wroclaw, Poland; agnieszka.orkusz@ue.wroc.pl; Tel.: +48-713680480

**Keywords:** child, diet, kindergarten, menus, nutritional errors

## Abstract

The evaluation of nutrition is an essential element of preventing chronic diseases and can be used to determine nutritional recommendations. A child spends about 7–8 h a day in a kindergarten; therefore, meals served there should be balanced appropriately to ensure the full psychophysical development of the young organism. At preschool age, children develop eating habits that can have life-long effects. Based on 10-day menus, the study aimed to estimate the energy and nutritional value of children’s diets at four randomly selected kindergartens in the Wroclaw district, Poland. In total, 80 menus were analyzed (40 for summer and 40 for autumn). The data from kindergartens were analyzed based on the Diet 6D computer program. Regardless of the kindergarten, the analyzed food rations showed irregularities related to excessive supplies (in reference to the dietary recommendations) of sucrose, fiber, salt, magnesium, and vitamin A. The preschool food rations did not cover demands with respect to PUFA n-3, PUFA-n-6, calcium, and vitamin D. The observed irregularities confirm the need to monitor the content of energy and nutrients in preschool menus to be able to correct any dietary errors.

## 1. Introduction

The proper nutrition of pre-school children guarantees their optimal health [1,2]. Because a child spends about 7–8 h a day in kindergarten, the food served there should be balanced appropriately to provide energy and nutrients in the right amounts and proportions [3]. The collective nutrition of children and adolescents within the education system must meet the appropriate requirements for given age groups, adhering to the current nutrition standards for the Polish population [4]. The following standards were adopted in the latest 2020 Polish nutrition standards: Estimated Energy Requirement (EER); Estimated Average Requirement (EAR); Recommended Dietary Allowance (RDA); Adequate Intake (AI), and Reference Intake ranges for macronutrients (RIs) [4]. In planning mass nutrition in kindergartens, the Recommended Dietary Allowance (RDA) level should be applied. This guarantees that the planned nutrition will cover the average demand of the group of preschoolers at the EAR level [5]. In addition, the Tolerable Upper Intake Level (UL) has been developed for vitamins and mineral ingredients. The UL is the maximum level of nutrient intake from all sources (food, drinking water, dietary supplements) that does not cause adverse health effects in almost all people in each group. Consumption of nutrients above this level may cause adverse health effects. The UL value is a guideline level that should not be pursued as a standard but should be observed to ensure that it is not exceeded [4]. A child receives at least 3 meals daily in kindergarten. Therefore, it is assumed that the daily preschool ration should cover 75% of energy and nutrient needs [2,5,6,7,8,9,10].

Proper nutrition in kindergarten is also crucial for shaping the correct eating habits of children and can prevent the later development of many diet-related diseases (obesity, type 2 diabetes, cardiovascular diseases) [11]. The statistics related to obesity among children and adolescents are worrying across the world, including Poland. Data from the World Health Organization indicate an upward trend in obesity among children aged 5–9 and 10–19 [12]. Over 10 years, boys showed an increase in obesity of 6.3% and 4.8%, respectively, while girls showed an increase of 3.0% and 1.7%. It is estimated that, by 2025, if the rate of increase in obesity continues to grow at a similar pace, about 268 million children aged 5–17 years will be overweight, of whom 91 million will be obese [13]. In 2025, 38 million children will have hepatic steatosis or a buildup of fat in the liver, 27 million will have hypertension, 12 million will suffer from impaired glucose tolerance, and 4 million will have type 2 diabetes [14]. A lot of studies show that obesity in childhood is strongly associated with obesity in adulthood [15,16,17,18]. Most children who gained excess weight before the age of 6 remain overweight during puberty [18]. It has been shown that the earlier the increase in body fat occurs, the greater the final weight of the child is [15,19].

Many authors point to improper nutrition of children in kindergartens in Poland [7,20,21,22,23,24,25,26,27,28,29,30,31]. Meals are improperly balanced, both in terms of energy and nutritional value. It also happens that they are prepared from low-quality ingredients. This situation may be due to the insufficient knowledge and skills of the people responsible for preschool nutrition, which suggests the need to implement nutritional education for staff [2]. Perhaps a regular assessment of menus would help to avoid basic mistakes that occur when composing them.

Based on 10-day menus, the study aimed at estimating the energy and nutritional value of children’s diets in four randomly selected kindergartens in the Wroclaw district, Poland.

## 2. Materials and Methods

### 2.1. Data

The research was conducted in 2019. The menus from the 4 randomly selected preschool institutions (designated as: A, B, C, D) located in the Wroclaw district were assessed. There were 5–7 pre-school groups in the facilities, to which children aged 4 to 6 were assigned. Each group consisted of about 25 children. The kindergartens had their own kitchens where meals were prepared (the services of catering companies were not used). The 10-day menus from the summer and autumn seasons were assessed in each of 4 kindergartens. In total, 80 menus were analyzed (40 menus for summer and 40 menus for autumn).

To assess the energy value and the content of nutrients in the food rations, such as proteins, fats, carbohydrates (including glucose, fructose, sucrose, lactose, starch), fiber, minerals (sodium (Na), potassium (K), calcium (Ca), phosphorus (P), magnesium (Mg), iron (Fe), zinc (Zn), copper (Cu), and manganese (Mn)), vitamins (thiamine (B_1_), riboflavin (B_2_), niacin (B_3_), pyridoxine (B_6_), and vitamins A, C, D, and E), cholesterol, and fatty acids (saturated, monounsaturated, polyunsaturated), the Diet 6D computer program [32] was implemented. The salt content was also calculated. The Diet 6D program has proved to be the best in Poland so far. It offers the possibility of calculating energy intake and as many as 89 nutrients. The Diet program contains a food composition database based on the Polish food composition tables. The portion size was estimated based on the number of products, meals, and drinks expressed in grams specified in the menus. The calculations considered losses resulting from technological processes. Therefore, for the energy value, total protein, fats, carbohydrates, calcium, and iron, losses equal to 10% were used, and for vitamins A and C, losses equivalent to 20% and 55%, respectively, were used [33]. Then all values for 10-day menus were totaled. In this way, for each kindergarten, the average values of energy and nutrients were estimated. Next, the obtained results were compared with the applicable standards and recommendations for children aged 4–6 years [4]. Energy standards were set at the level of average demand (Estimated Energy Requirement). The Reference Intake ranges, expressed as a percentage of the energy requirement, were defined for fat and carbohydrates. The standards for protein, selected minerals (calcium, phosphorus, magnesium, iron, zinc, copper), and vitamins (thiamine, riboflavin, niacin, pyridoxine, vitamins A and C) were set as per the Recommended Dietary Allowance. Standards for other nutrients (sodium, potassium, manganese, vitamins D and E, fiber, n-3 PUFA, and n-6 PUFA) were set as per the level of sufficient consumption (Adequate Intake) [4].

To assess the diet of children aged 4–6 years, with a bodyweight of 19 kg and moderate physical activity, a daily energy requirement of 1400 kcal was adopted in accordance with the standards [4]. For children up to the age of 9, the standards are the same for boys and girls. Their differentiation by gender is applied in the older age groups. The energy standards for children up to 6 years of age include the same levels of physical activity. The norms for energy, protein, and fat were established based on body weight, which is understood as a reference body weight value, not the actual weight of a child. The data from studies representative of the Polish population were used to determine the reference body weight values for preschool children (3–6 years) [34]. The reference body weight value for children aged 4–6 years, based on the median, is 19 kg. The energy and nutrient content of the preschool diet was compared to 75% of the daily norm for children aged 4–6 [2,5,6,7,8,9,10]. According to this, the recommended value for energy was 1050 kcal [4]. The consumption values differing by ±10% from the standard values were assumed to be correct. The content of minerals (Mg, Zn, Cu) and vitamins (A, B_6_) was also compared to 75% of the Tolerable Upper Intake Level [35]. Various groups of experts have established UL levels for individual vitamins and minerals. UL values proposed by the European Food Safety Authority were used in the study [35]. It was assumed that the children consumed all served food.

### 2.2. Statistical Analysis

Two-way variance analysis (ANOVA) was used to evaluate changes in energy value and nutrients in kindergarten menus. The effect of such factors as the kindergarten (A, B, C, D), the season (summer and autumn), and a couple of interactions between them were analyzed. When a significant main effect or interaction was identified, the mean values were further analyzed using the Tukey’s multiple range test. The results were presented as a mean ± standard deviation. The experimental data were analyzed using Statistica version 13.3 (StatSoft Inc., Kraków, Poland) [36]. Differences with a probability level <0.05 were considered significant.

### 2.3. Ethical Statement

Ethical review and approval were waived for this study as the research was based on an evaluation of the preschool menus based on the computer program Diet 6D.

## 3. Results

Based on the analysis of variance results, it was shown that for energy and nutrients (except for copper, manganese, cholesterol, and PUFA n-3), the main effect was significant for the type of kindergarten for the adopted significance level of *p* < 0.05. For most nutrients, the season factor was not found to be significant. A two-way interaction effect occurred only with carbohydrates and starch (Table 1).

Based on the results of this research, it was shown that the energy value of meals differed significantly depending on the kindergarten, both in summer and in autumn (Table 1). The energy value of meals served in summer was correct in kindergartens A, B and C, while in autumn, this was the case only in kindergartens A and B. The energy value of menus in the kindergarten D was higher than the Estimated Energy Requirement, regardless of the season (Table 2).

The protein content in the analyzed preschool rations ranged from 30.7 g to 52.3 g in summer and from 37.5 g to 55.4 g in autumn, respectively (Table 1). This amount of protein exceeded the recommended level of consumption (intake above 75% of RDA) by two or even three times (Table 2). The menus from kindergarten D had a significantly higher content of this nutrient compared to the other kindergartens (Table 2).

The food ration of the children who spend part of the day in kindergarten should provide 35.3 g of fat. In this study, this amount was exceeded by 15.4% in summer and 21.5% in autumn in kindergarten D (Table 2). In the remaining kindergartens, the fat content was lower than recommended. From the nutritional point of view, it is also important to identify the type of fatty acids in the diet. In each kindergarten, the children’s diet was characterized by too low n-3 and n-6 polyunsaturated fatty acid content. An excess of unsatisfactory saturated fatty acids was found in kindergartens A and D in both seasons (Table 2).

The carbohydrate content differed significantly between preschools. The menus were balanced in terms of carbohydrate content in kindergartens B and C in summer and A and B in autumn (Table 2). An excessive supply of carbohydrates in summer and autumn was recorded in kindergarten D (Table 2). In the assessment of the supply of added sugars (sucrose, fructose, glucose), it was assumed that they should constitute no more than 10% of the diet’s energy. Regardless of the kindergarten, the sucrose content exceeded the recommended intake (Table 2). The proportion of carbohydrates from each group in the children’s meals was inappropriate (Table 3). The children’s diet was characterized by a too low proportion of starch-containing products. The dietary fiber content was above the recommended level in each facility (Table 2).

The analyzed menus, compared to the norms, covered the children’s needs for minerals (such as sodium, potassium, calcium, phosphorus, magnesium, calcium, iron, zinc, copper, manganese) to varying degrees. Regardless of the kindergarten, a significant calcium inadequacy was noted in the studied menus (Table 2). Its content in summer ranged from 250.4 mg to 473.2 mg, and in autumn, from 364.0 mg to 622.3 mg (with the recommended standard of 750 mg) (Table 1).

The iron content in kindergartens B and C summer and autumn menus was also below 75% of the RDA. The daily supply of this mineral exceeded 75% of RDA in kindergarten D in autumn (Table 2). At the same time, it should be emphasized that UL standards for iron have not been determined [35]. The content of the remaining analyzed minerals (sodium, potassium, phosphorus, magnesium, zinc, copper, and manganese) in all the analyzed menus was too high according to Polish standards (RDA, AI) (Table 2). It should also be noted that the intake of magnesium was higher than 75% of the UL in the fall in the kindergartens A and B and in institution D in both seasons, and for vitamin A in kindergartens A and D. The UL levels for zinc, copper, and vitamin B_6_ were not exceeded.

In the analyzed preschool menus there were irregularities in the vitamin content. A vitamin D deficiency (intake lower than 75% of AI) and an excess of vitamins A, B_1_, B_2_, and B_6_ (intakes above 75% of RDA) were demonstrated in each preschool (Table 2). The supply of niacin and vitamins C and E varied depending on the kindergarten (Table 2).

In all menus, there were irregularities in the salt content. The content in preschool rations ranged from 232.5 g to 325.0 g, respectively (Table 1). This amount of salt was twice or even three times higher than the recommended intake level (Table 2).

The all-day food ration in the kindergarten consisted of 3 meals: breakfast, lunch, and afternoon tea. The intervals between meals were adequate at 4 and 3 h, respectively.

Regardless of the kindergarten, breakfast was usually served with wheat bread, butter, and a high-protein product of animal origin (cold cuts of meat, eggs, cheese) or an item with high carbohydrate content such as jam (most often strawberry). Children were also served milk with corn flakes or other cereals. To drink, tea, grain coffee, or cocoa were provided. The drinks were sweetened with sugar. Each breakfast was served with a piece of fruit (most often apples, bananas) or a vegetable (most often cucumbers or tomatoes).

Lunch always consisted of two dishes: soup and a second course. The most frequently served soups included tomato, potato, barley, or chicken, and cabbage. They were prepared based on meat and vegetable stock. They were not enriched with fresh dill or parsley. The second course usually consisted of a protein product of animal origin—usually meat, a bulk product—mainly potatoes and less often pasta or rice, as well as cooked or raw vegetables. Compote with sugar or fruit and vegetable drinks were usually served with lunch.

Afternoon tea usually consisted of products rich in carbohydrates (buns, jelly, bread with chocolate spread, honey) and a warm drink (tea, milk, or milk drinks with added sugar). Sometimes sandwiches were served (with pate, pork sirloin, sausage, or jam).

The meals were prepared in a variety of techniques. The products were fried, boiled, stewed, or baked. The meals were composed with contrasts in taste, smell, and color to encourage children to eat. Combining two dishes of a similar nature (sweet, sour, or similar in color and texture) was avoided during one meal. No seasonality of products was found in the menus. The disadvantage of the analyzed menus was the lack of whole grain products, the limited quantities of fish and legumes, and the servings of meat products with high fat content (mortadella, sausages, and cheese). Spices were added in limited amounts.

## 4. Discussion

The statistical analysis of the obtained results showed that the energy value of the studied menus differed regarding the season and the kindergarten. In autumn, a favorable tendency of an energy increase in menus was observed when the body’s energy expenditure related to the need to maintain the body temperature at about 37 °C increased, with a simultaneous decrease in the surrounding temperature. The energy values of the kindergarten meals, except for facility D and C (in autumn), were correct. Preschool meals should cover the energy and nutrient needs of children to a greater extent than home meals. However, they should not exceed the recommendations because they lead to, among other issues, excess weight and obesity. The results of this study do not differ from the data in the literature on the nutrition of children in kindergartens [20,23,27,37,38,39], which report too high energy supply as compared to the recommendations.

Kindergarten D also reported excess fat in the children’s diet, which probably came from chocolate and confectionery products, which provided 10 to 15 g of fat per 100 g of product [32] and were also a source of unfavorable saturated fatty acids. The consumption of products rich in fat, including saturated fatty acids, is associated with the risk of ischemic heart disease and the development of cancer, mainly colorectal and breast cancer [40,41,42].

The average protein content of the evaluated food rations from the analyzed kindergartens was more than double the RDA norm. Similar results in preschool diets were also obtained by the other authors [22,27,30,43]. Such a protein supply was determined by the high consumption of meat and processed meat products. So far, an UL standard has not been defined for protein but attempts are underway to determine the upper limit of consumption. However, it is known that high protein consumption may be associated with hypercalciuria, resulting in osteoporosis, acidosis, and an increased risk of kidney stones [4,44]. Excess dietary protein content in childhood contributes to an increased risk of obesity [45].

An analysis of the data in the literature shows that there is a lack of studies on the intake of specific groups of carbohydrates, including fructose, which is extremely important because of its negative impact on human health [46,47,48]. The increased consumption of products (sweetened soft drinks, candies) to which fructose is added (especially as a glucose–fructose syrup [4]) during the production process was observed. The highest fructose content was found in the meals of kindergarten A. Excessive fructose intake is believed to be one of the factors responsible for the steady increase in excess weight and obesity problems in modern societies [49]. This is especially true in children and adolescents [50].

One of the tools to support the improvement of the nutrition of children and adolescents in educational institutions was the Regulation of the Minister of Health of 26 July 2016 on the groups of food intended for sale to children and adolescents within the educational system and the requirements for food served as part of the collective nutrition of children and adolescents in educational settings (Journal of Laws of 2016, item 1154) [51]. The provisions of the regulation covered, among others, requirements for collective nutrition in kindergartens, school establishments, boarding schools, and boarding houses. The regulation requires the inclusion of more vegetables and fruit so that they are present every day in each meal, with the inclusion of more fish in the weekly diet while limiting the addition of sugar in drinks prepared onsite to a maximum of 10 g per 250 mL of drink. This was designed to enable easier compliance with the standards’ recommendation to limit sugars (sucrose, fructose, glucose) added to food during its manufacture and production, which should provide no more than 10% of energy.

Despite the above-mentioned regulation, in each kindergarten the amount of sugar added to food during its production process exceeded recommendations. Sucrose content ranged from 31.6 g to 61.4 g, corresponding to 120% and 234.0% of the standard value, which is a disturbing phenomenon. It is well known that an excessive intake of sucrose and simple sugars contributes to the occurrence of many chronic non-communicable diseases, including excess weight and obesity problems, insulin resistance, diabetes, metabolic syndrome, tooth decay, cancer, and non-alcoholic fatty liver disease [52,53,54,55]. Other authors have also noted high sucrose content in the diet of preschool children [24,27].

It should be noted that from 1 January 2021, regulations entered into force in Poland introducing taxes on sweetened and energy drinks (ACT of 14 February 2020 on the amendment of certain acts in connection with the promotion of pro-health consumer choices) [56]. Perhaps this regulation will reduce the sale and consumption of sweetened beverages and improve the health indicators of the Polish society.

The mineral content of the study rations varied widely. In all the evaluated dietary rations from the studied kindergartens, the calcium content was too low compared to the standard. The low supply of calcium in the analyzed dietary rations was caused by the low supply of milk and milk products (e.g., cottage cheese, kefir, buttermilk), which represent the main sources of calcium in the diet. Adequate calcium content is particularly desirable in the diet of small children because calcium is an important building material for bones and teeth [57,58]; it is also involved in muscle contraction, blood coagulation, conduction of nerve impulses, activation of enzymes, and the reduction in the permeability of cell membranes, as well as supporting the proper functioning of the cardiovascular system. Calcium inadequacy in children’s diets is a common problem [28,29,30,59,60,61]. The utilization of calcium from the diet is hindered by a too-high phosphorus content [62]. An adequate calcium/phosphorus ratio is a very significant factor in calcium phosphate metabolism. A too low calcium/phosphorus ratio (indicating a high dietary phosphorus intake) stimulates parathormone secretion and enhances calcium resorption from the bones. The calcium-to-phosphorus ratio was abnormal in the children’s diets, ranging between 0.5 to 0.6 regardless of the preschool institution and a season.

It should also be mentioned that the factors increasing calcium absorption include, among others, vitamin D, the amount of which in the analyzed diets was significantly below the norm. Insufficient intake of calcium and vitamin D raises serious concerns about the proper condition of children’s skeletons. The results of this research indicate that it is necessary to educate the employees of preschool institutions responsible for children’s nutrition. Social campaigns promoting the importance of calcium intake and indicating food products and their quantities needed to meet the child’s demand for this mineral may be a way to draw public attention to the consequences of calcium deficiency in children.

Based on the research results, it was found that, regardless of the kindergarten, the sodium content in the children’s diet was very high (intake higher than 75% of AI). Such high sodium intake indicates the need to limit the addition of salt to dishes and the elimination of processed products with a high salt content (e.g., cold meat products). The results of sodium consumption converted into table salt exceeded the recommendations by more than 2–3 times. There is no doubt that salt consumption in Poland should be reduced [2,3,6]. It is advisable to develop proper eating habits from an early age to reduce the risk of developing cardiovascular diseases, arterial hypertension, and cancer of the stomach and throat at a later age [63,64]. Many studies report excessive sodium and table salt intake in children’s diets, suggesting that it is a common phenomenon [30,59,65,66,67].

Regardless of the kindergarten, the analyzed food rations also contained too high intakes of potassium, copper, manganese, and magnesium compared to the Polish norms. It should be noted that EFSA experts concluded that there is currently insufficient scientific research on the negative effects of the excess of sodium, potassium, and manganese on the human body [35]. Consequently, it is currently impossible to establish ULs for these components. The UL for copper intake was not exceeded. In this situation, the intake of potassium, copper, and manganese is satisfactory. The intake of magnesium was higher than 75% of UL. Doses of magnesium above 250 mg/day have laxative properties [4].

The analyzed food rations covered the children’s iron needs to varying degrees. In kindergartens B and C, iron deficiency was demonstrated, while in kindergarten D an excess was found. At the same time, it should be emphasized that UL standards for iron have not been determined [35]. It is well known that iron deficiency promotes the development of anemia [68] and may reduce a child´s immunity [69]. At the same time, its excess increases the risk of cancer, gastric disorders, atherosclerosis, strokes, and Alzheimer’s and Parkinson’s disease [70,71,72]. Literature data also show large variations in iron consumption among children, usually insufficient [28,30,39,67,73] or exceeding the norm [22,43].

Regardless of the kindergarten, the children’s food rations showed an excess of vitamins A, B_1_, B_2_, B_6_, and C (except for kindergarten B) and vitamin D deficiency. The study results do not differ from the results of the studies by the other authors who found excessive intake of vitamin A in preschool food rations compared to the norms [28,30,43]. An excessive supply of vitamin A manifests itself in irritability, headaches, loss of appetite, dry skin, and digestive tract disorders, which are accompanied by enlargement of the liver and spleen [1]. Since the UL values have not been established for vitamins B_1_, B_2_ and C and the UL value standard for vitamin B_6_ was not exceeded, their intake level can be assessed positively.

Children’s food rations were characterized by a deficient supply of vitamin D, whose deficiency may result in the negative calcium balance and impaired bone mineralization, resulting in rickets in children [74]. Deficiency of this vitamin was due to a very low intake of fish, which was served sporadically, regardless of the preschool. Vitamin D is found in smaller amounts in butter, yellow cheese, egg yolk, and meat, which were served to the children in meals. However, with the deficient intake of fish, the other listed sources of the vitamin did not cover the children’s vitamin D requirements.

Numerous studies indicate irregularities in the nutrition of children in kindergartens. The most frequent recurring problem is an excessive supply of fat, vitamin A, salt, and calcium, and vitamin D deficiency, which is also confirmed by the results of this study.

It should be mentioned that the data on the energy and nutritional value of children diet were obtained from the tables of composition and nutritional value [32]. The tables’ values are averaged data, so the energy and nutrient content of a product consumed by children may differ from the values given in the tables. The content can depend on many factors, both genetic and environmental. The limitation of this study is the lack of data regarding the real food consumption of children, food waste on the plate, and the real amount of salt added to dishes. Consequently, the estimated content of energy and nutrients in the daily food rations of children may differ from their actual consumption. Thus, the observed deficiencies of the selected nutrients in the children’s diets are hazardous, as their actual consumption may be even lower. It is essential to be aware that preschool nutrition represents only a part of the diet. It is not known what foods products the children received at home and whether their parents introduced supplements into their diet. It is worth mentioning that the menu data were acquired through 2019 only. However, they are still valid, as during 2020 and 2021 there were lockdowns, and kindergartens were closed. Therefore, there were more important issues to combat than kindergarten menu adjustment to the proper nutrition schemes.

## 5. Conclusions

The adequate nutrition of children is necessary to ensure their proper development. The evaluation of preschool food rations is important to prevent nutrition errors in this group. The study results indicate that the analyzed preschools did not fully implement the requirements set out in the Regulation of the Ministry of Health of 26 July 2016 [51]. Not every kindergarten meal included vegetable or fruit. More than two portions of fried food were served per week. The children’s diet did not contain nuts. Fish was served sporadically. One of the disadvantages of the analyzed menus was the presence of sugary foods. Chocolate, honey, and cornflakes with added sugar were often served for breakfast. The afternoon snack was served with confectionery products such as buttered buns and puff pastries. As is commonly known, sweets in a child’s diet should be limited to a minimum, and such an institution as a kindergarten should provide children with proper models concerning nutrition. No seasonality of products was noticed in the menus. The same fruit and vegetables were served in summer and autumn. The assessment of menus, regardless of the kindergarten, showed that they were arranged incorrectly, which indicates the need for modification of their composition. Therefore, it is necessary to carry out nutritional education among kindergarten staff. To ensure the health of Polish children and thus the health of future society, it is necessary to correct dietary mistakes as soon as possible, since diet-related diseases may develop from childhood.

## Figures and Tables

**Table 1:** Mean values (± standard deviation) of energy value and nutrient content in preschool menus.

Energy and Nutrients	Season (S)	Kindergarten (K)				Significant Effects		
		A	B	C	D	K	S	K × S
Energy (kcal)	Summer	945.4 ^a^ ± 205.7	1024.4 ^a^ ± 148.9	1059.8 ^a^ ± 138.3	1397.3 ^b^ ± 13.5	***	**	ns
	Autumn	1121.8 ^a^ ± 116.7	1029.4 ^a^ ± 108.5	1217.7 ^a^ ± 238.7	1397.1 ^b^ ± 6.6			
Protein (g)	Summer	30.7 ^a^ ± 6.7	35.8 ^a^ ± 3.6	36.2 ^a^ ± 8.0	52.3 ^b^ ± 9.6	***	*	ns
	Autumn	40.5 ^a^ ± 9.6	37.5 ^a^ ± 4.8	39.1 ^a^ ± 10.0	55.4 ^b^ ± 14.4			
Fat (g)	Summer	29.4 ^ab^ ± 13.3	24.6 ^a^ ± 4.4	23.6 ^a^ ± 4.5	40.7 ^b^ ± 14.5	***	ns	ns
	Autumn	30.1 ^ab^ ± 7.5	29.6 ^ab^ ± 7.5	22.8 ^a^ ± 7.8	42.8 ^b^ ± 12.9			
Carbohydrates (g)	Summer	146.7 ^a^ ± 27.3	171.5 ^ab^ ± 33.5	183.7 ^ab^ ± 29.8	212.6 ^b^ ± 33.1	***	ns	*
	Autumn	181.0 ^ab^ ± 26.7	159.4 ^a^ ± 24.4	222.2 ^b^ ± 54.8	205.8 ^ab^ ± 28.4			
Sodium (mg)	Summer	1763.3 ^a^ ± 332.5	1747.5 ^a^ ± 382.2	2100.5 ^a^ ± 422.6	2261.3 ^a^ ± 755.9	**	ns	ns
	Autumn	2172.6 ^ab^ ± 386.7	1756.5 ^a^ ± 282.0	1974.3 ^ab^ ± 378.0	2448.2 ^b^ ± 574.5			
Potassium (mg)	Summer	1928.1 ± 374.5	1652.0 ± 350.7	2085.4 ± 455.0	2070.4 ± 589.4	**	ns	ns
	Autumn	2337.7 ^a^ ± 550.3	1577.2 ^b^ ± 321.7	1988.1 ^ab^ ± 400.0	2313.3 ^ab^ ± 535.5			
Calcium (mg)	Summer	250.4 ^a^ ± 102.6	401.9 ^ab^ ± 130.3	373.2 ^ab^ ± 107.4	473.2 ^b^ ± 201.4	***	ns	ns
	Autumn	364.0 ^a^ ± 148.9	380.6 ^a^ ± 110.2	365.9 ^a^ ± 111.3	622.3 ^b^ ± 222.8			
Phosphorus (mg)	Summer	476.6 ^a^ ± 121.3	652.8 ^ab^ ± 82.2	653.0 ^ab^ ± 163.5	821.2 ^b^ ± 171.1	***	**	ns
	Autumn	643.8 ^a^ ± 191.7	679.4 ^a^ ± 141.3	675.3 ^a^ ± 123.8	1022.6 ^b^ ± 257.2			
Calcium/Phosphorus	Summer	0.5 ± 0.2	0.6 ± 0.2	0.6 ± 0.1	0.6 ± 0.1	ns	ns	ns
	Autumn	0.6 ± 0.1	0.6 ± 0.1	0.5 ± 0.1	0.6 ± 0.2			
Magnesium (mg)	Summer	142.0 ± 35.3	170.4 ± 30.0	182.2 ± 46.5	196.8 ± 51.0	**	*	ns
	Autumn	194.9 ± 49.7	188.9 ± 57.3	179.5 ± 37.5	259.5 ± 113.8			
Iron (mg)	Summer	5.3 ± 0.7	5.9 ± 0.8	6.2 ± 1.5	7.3 ± 1.3	*	*	ns
	Autumn	7.0 ^ab^ ± 1.8	6.2 ^a^ ± 1.4	6.5 ^a^ ± 1.1	9.8 ^b^ ± 1.7			
Zinc (mg)	Summer	3.9 ^a^ ± 0.6	5.1 ^ab^ ± 0.5	4.7 ^ab^ ± 1.1	6.1 ^b^ ± 1.7	***	*	ns
	Autumn	5.4 ± 1.9	5.4 ± 1.3	5.0 ± 1.0	6.8 ± 1.7			
Copper (mg)	Summer	0.7 ± 0.1	0.9 ± 0.2	0.8 ± 0.2	0.9 ± 0.3	ns	ns	ns
	Autumn	0.9 ± 0.2	0.9 ± 0.2	0.8 ± 0.1	1.0 ± 0.3			
Manganese (mg)	Summer	2.3 ± 0.3	2.7 ± 0.6	2.5 ± 0.8	2.8 ± 1.2	ns	ns	ns
	Autumn	2.8 ± 1.4	3.0 ± 0.4	2.5 ± 0.5	3.5 ± 1.7			
Vitamin B_1_ (mg)	Summer	0.6 ± 0.2	0.6 ± 0.2	0.5 ± 0.2	0.8 ± 0.3	*	ns	ns
	Autumn	0.8 ± 0.3	0.6 ± 0.1	0.7 ± 0.2	0.7 ± 0.3			
Vitamin B_2_ (mg)	Summer	0.6 ^a^ ± 0.1	0.9 ^ab^ ± 0.3	0.8 ^ab^ ± 0.2	1.0 ^b^ ± 0.2	***	*	ns
	Autumn	0.9 ^a^ ± 0.3	0.9 ^ab^ ± 0.1	0.8 ^a^ ± 0.3	1.2 ^b^ ± 0.3			
Niacin (mg)	Summer	7.8 ± 3.3	5.4 ± 1.6	8.2 ± 3.8	10.4 ± 3.6	***	ns	ns
	Autumn	9.7 ^ab^ ± 2.5	5.4 ^a^ ± 1.6	8.4 ^ab^ ± 2.9	12.0 ^b^ ± 8.5			
Vitamin C (mg)	Summer	77.5 ± 27.6	51.9 ± 12.6	67.0 ± 29.8	48.2 ± 20.6	***	ns	ns
	Autumn	98.1 ^a^ ± 70.0	37.4 ^b^ ± 16.4	62.0 ^ab^ ± 22.9	55.0 ^ab^ ± 22.6			
Vitamin A (µg)	Summer	857.4 ± 309.8	689.6 ± 281.1	758.9 ± 426.2	827.9 ± 277.4	*	ns	ns
	Autumn	975.3 ^ab^ ± 370.7	631.2 ^a^ ± 346.1	801.6 ^ab^ ± 289.0	1210.3 ^b^ ± 702.4			
Vitamin E (mg)	Summer	4.0 ± 1.0	3.7 ± 0.7	3.4 ± 0.7	4.7 ± 1.0	***	ns	ns
	Autumn	4.0 ^a^ ± 0.7	4.3 ^ab^ ± 1.6	3.7 ^a^ ± 1.1	5.5 ^b^ ± 1.5			
Vitamin B_6_ (mg)	Summer	1.0 ± 0.2	0.8 ± 0.2	1.1 ± 0.3	1.2 ± 0.4	***	ns	ns
	Autumn	1.2 ^a^ ± 0.2	0.8 ^b^ ± 0.3	1.0 ^ab^ ± 0.3	1.2 ^a^ ± 0.5			
Vitamin D (µg)	Summer	0.8 ± 0.4	1.0 ± 0.7	0.8 ± 0.6	1.8 ± 0.7	*	ns	ns
	Autumn	1.1 ± 0.8	1.2 ± 1.0	0.8 ± 0.4	1.3 ± 0.9			
Glucose (g)	Summer	10.6 ^a^ ± 2.8	5.3 ^b^ ± 1.4	7.5 ^ab^ ± 2.3	6.0 ^b^ ± 3.5	***	ns	ns
	Autumn	10.4 ^a^ ± 3.2	5.0 ^b^ ± 2.2	7.1 ^ab^ ± 2.2	5.8 ^b^ ± 1.8			
Sucrose (g)	Summer	31.6 ± 8.3	40.1 ± 17.4	51.6 ± 13.7	34.1 ± 17.9	**	*	ns
	Autumn	40.1 ± 7.2	43.0 ± 12.4	61.4 ± 33.2	43.5 ± 12.2			
Fructose (g)	Summer	13.3 ^a^ ± 3.4	6.9 ^b^ ± 2.7	9.2 ^ab^ ± 2.6	6.5 ^b^ ± 3.2	***	ns	ns
	Autumn	12.2 ^a^ ± 4.7	6.8 ^b^ ± 2.0	8.7 ^ab^ ± 4.1	7.7 ^ab^ ± 2.6			
Lactose (g)	Summer	2.7 ^a^ ± 2.6	8.1 ^b^ ± 3.9	6.2 ^ab^ ± 4.8	7.7 ^b^ ± 2.7	***	ns	ns
	Autumn	4.9 ^a^ ± 3.8	8.6 ^ab^ ± 2.5	6.9 ^ab^ ± 4.1	10.3 ^b^ ± 3.7			
Starch (g)	Summer	62.0 ^a^ ± 12.6	89.1 ^ab^ ± 45.5	82.7 ^a^ ± 21.4	124.6 ^b^ ± 24.1	***	ns	*
	Autumn	76.3 ± 27.3	76.0 ± 18.3	106.2 ± 34.8	100.6 ± 14.9			
Fibre (g)	Summer	14.7 ± 2.3	13.2 ± 2.5	16.1 ± 4.7	14.5 ± 3.1	*	ns	ns
	Autumn	17.6 ± 2.7	12.8 ± 2.1	16.3 ± 2.6	16.7 ± 6.4			
Cholesterol (mg)	Summer	142.6 ± 73.0	165.4 ± 125.6	120.4 ± 88.2	184.2 ± 92.6	ns	ns	ns
	Autumn	187.2 ± 169.9	163.9 ± 101.4	108.3 ± 78.1	183.6 ± 67.5			
SFA (g)	Summer	15.2 ^ab^ ± 8.8	11.6 ^ab^ ± 2.4	10.6 ^a^ ± 2.4	20.4 ^b^ ± 10.3	***	ns	ns
	Autumn	13.9 ^ab^ ± 5.0	13.3 ^ab^ ± 3.3	10.7 ^a^ ± 5.4	21.0 ^b^ ± 7.5			
MUFA (g)	Summer	9.6 ^ab^ ± 3.8	7.7 ^a^ ± 1.7	7.2 ^a^ ± 2.2	12.7 ^b^ ± 4.6	***	ns	ns
	Autumn	10.7 ^ab^ ± 3.4	9.7 ^ab^ ± 3.2	6.8 ^a^ ± 2.7	13.2 ^b^ ± 4.8			
PUFA (g)	Summer	2.2 ^a^ ± 0.4	3.2 ^ab^ ± 0.5	3.6 ^ab^ ± 3.0	4.4 ^b^ ± 0.9	***	ns	ns
	Autumn	2.8 ± 0.6	4.1 ± 1.5	3.5 ± 1.6	4.6 ± 1.1			
PUFA/SFA	Summer	0.2 ± 0.1	0.3 ± 0.1	0.4 ± 0.6	0.3 ± 0.2	*	ns	ns
	Autumn	0.2 ± 0.1	0.3 ± 0.2	0.4 ± 0.4	0.2 ± 0.1			
PUFA n-3 (g)	Summer	0.4 ± 0.1	0.4 ± 0.1	0.6 ± 0.5	0.6 ± 0.2	ns	ns	ns
	Autumn	0.5 ± 0.1	0.5 ± 0.4	0.5 ± 0.3	0.7 ± 0.3			
PUFA n-6 (g)	Summer	1.8 ^a^ ± 0.4	2.8 ^ab^ ± 0.4	3.0 ^ab^ ± 2.6	3.8 ^b^ ± 0.8	***	ns	ns
	Autumn	2.3 ± 0.6	3.6 ± 1.4	2.9 ± 1.5	3.8 ± 1.0			
PUFA n-6/n-3	Summer	4.4 ± 1.0	7.4 ± 1.1	4.6 ± 1.4	7.1 ± 2.7	***	ns	ns
	Autumn	4.7 ^a^ ± 1.8	8.6 ^b^ ± 4.3	6.1 ^ab^ ± 2.8	5.7 ^ab^ ± 2.2			
Salt (g)	Summer	4.4 ± 0.8	4.4 ± 1.0	5.3 ± 1.1	5.7 ± 1.9	**	ns	ns
	Autumn	5.4 ^ab^ ± 1.0	4.4 ^a^ ± 0.7	4.9 ^ab^ ± 0.9	6.1 ^b^ ± 1.4			

Significant effects: *** *p* < 0.001; ** 0.001 ≤ *p* < 0.01; * 0.01 ≤ *p* < 0.05; ns—not significant; K—kindergarten; S—season. a–b: Means. Different letters in the same row indicate differences at p < 0.05 in view of the kindergarten. SFA—saturated fatty acids, MUFA—monounsaturated fatty acids, PUFA—polyunsaturated fatty acids.

**Table 2:** Energy and nutrients in preschool food rations constituting 75% of the Polish norms and recommendations and the UL established by the EFSA.

Energy and Nutrients	Norm	75% of the Daily Requirement	Season	Norm Realization (%)			
				Kindergarten A	B	C	D
Energy (kcal)	1400 ^1^	1050 ^1^	Summer	90.0 ^a^	97.6 ^a^	100.9 ^a^	133.1 ^b^
			Autumn	106.8 ^a^	98.0 ^a^	116.0 ^a^	133.1 ^b^
Protein (g)	21 ^2^	15.8 ^2^	Summer	195.0 ^a^	227.4 ^a^	229.7 ^a^	332.3 ^b^
			Autumn	257.2 ^a^	238.2 ^a^	248.1 ^a^	351.9 ^b^
Fat (g)	47 ^3^	35.3 ^3^	Summer	83.4 ^ab^	69.7 ^a^	66.9 ^a^	115.4 ^b^
			Autumn	85.3 ^ab^	84.1 ^ab^	64.6 ^a^	121.5 ^b^
Carbohydrates (g)	227.5 ^3^	170.6 ^3^	Summer	86.0 ^a^	100.5 ^ab^	107.7 ^ab^	124.6 ^b^
			Autumn	106.1 ^ab^	93.4 ^a^	130.2 ^b^	120.6 ^ab^
Sodium (mg)	1000 ^4^	750 ^4^	Summer	235.1	233.0	280.1	301.5
			Autumn	289.7 ^ab^	234.2 ^a^	263.2 ^ab^	326.4 ^b^
Potassium (mg)	1100 ^4^	825 ^4^	Summer	233.7	200.2	252.8	251.0
			Autumn	283.4 ^a^	191.2 ^b^	241.0 ^ab^	280.^ab^
Calcium (mg)	1000 ^2^	750 ^2^	Summer	33.4 ^a^	53.6 ^ab^	49.8 ^ab^	63.1 ^b^
			Autumn	48.5 ^a^	50.7 ^a^	48.8 ^a^	83.0 ^b^
Phosphorus (mg)	500 ^2^	375 ^2^	Summer	127.1 ^a^	174.1 ^ab^	174.1 ^ab^	219.0 ^b^
			Autumn	171.7 ^a^	181.2 ^a^	180.1 ^a^	272.7 ^b^
Magnesium (mg)	130 ^2^/250 ^5^	97.5 ^2^/187.5 ^5^	Summer	145.6/75.7	174.8/90.9	186.9/97.2	201.8/105.0
			Autumn	199.9/104.0	193.7/100.8	184.1/95.7	266.1/138.4
Iron (mg)	10.0 ^2^	7.5 ^2^	Summer	70.0	78.9	82.2	97.4
			Autumn	93.5 ^ab^	82.7 ^a^	86.8 ^a^	130.2 ^b^
Zinc (mg)	5.0 ^2^/10.0 ^5^	3.8 ^2^/7.5 ^5^	Summer	104.8/52.4	124.8/68.3	124.8/62.4	161.5/80.8
			Autumn	144.0/72.0	136.6/71.3	133.0/66.5	180.5/90.3
Copper (mg)	0.4 ^2^/2.0 ^5^	0.3 ^2^/1.5 ^5^	Summer	228.9/46.0	281.8/56.6	274.8/54.7	291.5/58.0
			Autumn	285.7/57.3	283.5/56.7	268.0/53.3	331.2/66.0
Manganese (mg)	1.5 ^4^	1.1 ^4^	Summer	203.3	242.5	223.4	245.6
			Autumn	244.2	265.4	223.6	305.3
Vitamin B_1_ (mg)	0.6 ^2^	0.5 ^2^	Summer	130.7	122.1	119.0	173.6
			Autumn	169.1	122.1	145.0	158.7
Vitamin B_2_ (mg)	0.6 ^2^	0.5 ^2^	Summer	143.0 ^a^	203.5 ^ab^	179.3 ^ab^	225.0 ^b^
			Autumn	195.8 ^a^	200.3 ^ab^	184.8 ^a^	267.3 ^b^
Niacin (mg)	8.0 ^2^	6.0 ^2^	Summer	130.7	89.9	136.6	173.4
			Autumn	162.3 ^ab^	90.5 ^a^	140.1 ^ab^	199.1 ^b^
Vitamin C (mg)	50.0 ^2^	37.5 ^2^	Summer	206.7	138.5	178.7	128.4
			Autumn	261.5 ^a^	99.7 ^b^	165.2 ^ab^	146.7 ^ab^
Vitamin A (µg)	450 ^2^/1100 ^5^	337.5 ^2^/825 ^5^	Summer	254.0/103.9	204.3/83.6	224.9/92.0	245.3/100.4
			Autumn	289.0 ^ab^/118.2	187.0 ^a^/76.5	237.5 ^ab^/97.2	358.6 ^b^/146.7
Vitamin E (mg)	6.0 ^4^	4.5^4^	Summer	88.0	83.1	75.0	104.9
			Autumn	87.7 ^a^	94.4 ^ab^	82.1 ^a^	123.0 ^b^
Vitamin B_6_ (mg)	0.6 ^2^/7.0 ^5^	0.5 ^2^/5.3 ^5^	Summer	223.3/19.1	180.9/15.4	238.2/20.4	263.3/22.5
			Autumn	273.6 ^a^/23.4	169.7 ^b^/14.5	223.1 ^ab^/19.1	275.0 ^a^/23.6
Vitamin D (µg)	15.0 ^4^	11.3 ^4^	Summer	6.9	8.5	7.2	15.6
			Autumn	9.9	10.5	7.2	11.7
Glucose (g)	* 10% energy value		Summer	40.2 ^a^	20.3 ^b^	28.7 ^ab^	23.0 ^b^
			Autumn	39.8 ^a^	19.2 ^b^	26.9 ^ab^	21.9 ^b^
Sucrose (g)		26.3	Summer	120.2	152.8	196.7	130.0
			Autumn	152.8	163.7	234.0	165.8
Fructose (g)			Summer	50.6 ^a^	26.3 ^b^	35.2 ^ab^	24.7 ^b^
			Autumn	46.3 ^a^	25.9 ^b^	33.1 ^ab^	29.1 ^ab^
Fiber (g)	14 ^4^	10.5 ^4^	Summer	140.4	125.3	153.2	137.6
			Autumn	167.6	122.1	154.8	158.6
Cholesterol (mg)	300	225	Summer	63.4	73.5	53.5	81.9
			Autumn	83.2	72.9	48.1	81.6
SFA (g)	15.6	11.7	Summer	130.1 ^ab^	98.9 ^ab^	90.5 ^a^	174.7 ^b^
			Autumn	119.2 ^ab^	113.8 ^ab^	91.3 ^a^	179.8 ^b^
PUFA n-3 (g)	1.0 ^4^	0.8 ^4^	Summer	54.8	49.4	82.4	76.3
			Autumn	63.5	68.4	64.6	95.5
PUFA n-6 (g)	6.2 ^4^	4.7 ^4^	Summer	39.0 ^a^	60.1 ^ab^	64.2 ^ab^	81.2 ^b^
			Autumn	49.0	77.1	61.0	81.8
Salt (g)	2.5	1.9	Summer	234.7	232.5	279.3	301.1
			Autumn	289.1 ^ab^	233.5 ^a^	262.8 ^ab^	325.0 ^b^

* 10% energy value of food ratio; ^1^ Estimated Energy Requirement (EER); ^2^ Recommended Dietary Allowance (RDA); ^3^ Reference Intake ranges (RIs); ^4^ Adequate Intake (AI); ^5^ Tolerable Upper Intake Level (UL). SFA—saturated fatty acids, MUFA—monounsaturated fatty acids, PUFA—polyunsaturated fatty acids. a–b: Means. Different letters in the same row indicate differences at *p* < 0.05 in view of the kindergarten.

**Table 3:** Percentage of carbohydrate groups in total carbohydrate content in kindergarten menus.

Type of Carbohydrates	Season	Kindergatren			
		A	B	C	D
Glucose	Summer	7.2	3.1	4.1	2.8
	Autumn	5.8	2.9	3.2	2.8
Sucrose	Summer	21.5	23.4	28.2	16.1
	Autumn	22.2	27.0	27.6	21.1
Fructose	Summer	9.1	4.0	5.0	3.1
	Autumn	6.7	4.3	3.9	3.7
Lactose	Summer	1.8	4.8	3.4	3.6
	Autumn	2.7	5.4	3.1	5.0
Starch	Summer	42.3	52.0	45.0	58.6
	Autumn	42.2	47.7	47.8	48.9

## Data Availability

Not applicable.
